# Time response enhancement for variable speed drive systems by using five-level cascade four quadrant chopper in dc-link

**DOI:** 10.1016/j.heliyon.2020.e04739

**Published:** 2020-08-16

**Authors:** Safwan Nadweh, Ola Khaddam, Ghassan Hayek, Bassan Atieh, Hassan Haes Alhelou

**Affiliations:** a Electrical Power Engineering, Tishreen University, Lattakia, Syria; b Mechatronics Engineering, Manara University, Lattakia, Syria

**Keywords:** Electrical engineering, Energy, Industrial engineering, Applied computing, Information systems, Variable speed drive system VSDS, Four-quadrant chopper, Power quality, Cascade multi-level converters, DC-link, Harmonic mitigation

## Abstract

This research introduces a filtering circuit design integrated with the DC-link of a medium power variable speed drive (VSD) system. The designed filtering circuit employs modern power electronic circuits to improve time response characteristics in both grid and dc-link sides. Depending on the widely used multi-level converters, a conventional three-level H-bridge four-quadrant chopper scheme was developed into a cascade five-level H-bridge four-quadrant chopper scheme. The time response of the proposed system was performed with both choppers, and results showed that voltage drop on cascade chopper transistors was reduced to half in comparison with voltage drop on conventional four-quadrant chopper transistors. Moreover, the sharp fluctuations in system's waves were mitigated; consequently, time response characteristics in both steady and transient states were remarkably improved. The total harmonic distortion factor THD% of the input current and voltage was reduced to 26.2% and 2.43% respectively and the ripple factor RF for DC-link current was reduced to 0.196.

## 1 Introduction

The electrical drive is one of the mainstays of the international economy; it provides comfort and prosperity in modern daily life and improve living standards. Drive systems have many important advantages, such as increased production, reduced costs, high efficiency, and optimum investment of energy. DC motors have been able to achieve satisfactory performance at high torque and speed requirements and conform to the accurate industrial standards. The same is true for AC motors such as synchronous and induction motors. DC motors are controlled by changing the current and voltage, while Induction motors are controlled by changing the current and frequency. When compared to DC motors, AC motors cost less, require less maintenance, and are more robust in abnormal surroundings. Moreover, AC motors can work at voltage values of up to 25KV, and can be designed with capacities greater than 2000KW and speeds up to 5000 r.p.m. Both AC and DC drives have the same operational principle. When AC motors are used in VSD systems, voltage is firstly rectified to produce DC voltage, then the rectified DC voltage is converted into AC voltage with the required modulations, which is achieved using power electronics such as the different topologies of converters. One significant characteristic of electronics is the ability to produce inconstant voltage and frequency from constant voltage and frequency. In previous years, drive converters were designed using Silicon Controlled Rectifiers SCR_S_. Nowadays, AC drives use a series of transistors to convert DC to AC with a variable frequency. Thus, the output current feeds the motor with the required frequency and voltage to produce the required motor speed. The evolution of power electronics and digital control circuits have contributed to a much easier control process, and became an alternative to DC motors’ driving circuits in industrial applications that require variable speed motors, especially in explosive places, such as mines and chemical industries. In addition, they are suitable for pumps, fans, compressors, and electrical trains. Many applications can make use of variable speed systems, such as conveyors, elevators, and centrifugal machines.

About 60% of the overall produced power is universally consumed by various types of electrical motors. In order to achieve greater energy savings in speed-controlled electric motors, variable speed drive systems are used considering changing flow requirements. Most important concerns in energy field these days are the ever-rising prices and the limited resources of energy, which justifies the need to improve the efficiency of driving systems as they contribute to the reduction of energy consumption. Although using drive systems contributes to a high efficiency performance and great energy savings, these systems can cause several power quality problems, which prompted concerns about power quality improving issues such as power factor increasing, losses reduction, and total harmonic distortion factor THD% reduction. The distribution electrical networks suffer from high compatibility components when operating under nonlinear load condition, which leads to major problems such as, power factor correction capacitor breakdown, resonance, and equipment overheating that leads to fast aging of these equipment [1]. The grid side currents in VSD system contain many harmonic components due to the presence of nonlinear elements, such as rectifiers and other power electronics schemes [2]. The THD% factor of variable speed systems input currents ranges in value from 100% to 140%, taking into account that the fifth order harmonic currents comprise 70% of the fundamental component and the seventh order harmonic currents comprise 58% of the fundamental component, while 11th, 12th order harmonic currents have much less effects on THD% [2, 3]. Harmonics can be expressed using Eq. (1) as below [3].

(1) h=n p∓1

where:

h: Generated harmonics.

n: Integer number.

P: The number of used rectifier pulses.

In order to attain minimum harmonic distortion when designing a variable speed drive system, we have two options: either using non-linear equipment with a little harmonic distortion, or installing harmonic mitigation equipment on the plant's input. The main purpose of all power quality solutions is to avoid the so-called electromagnetic interference (EMI) [4]. This can be achieved by reducing emissions from devices as much as possible, increasing equipment immunity and trying to reduce disturbances transmission into high-sensitivity receiving devices [5].

Harmonic mitigation methods can be divided into two groups, one of these groups is concerned with voltage harmonics reduction, and the other is concerned current harmonics reduction. These methods are mainly applied to maintain the standard THD% limits and avoid interference [6]. The AC current drawn by charging capacitors in these systems is the main cause of harmonics. Harmonics mitigation methods focus on reducing the capacitance of the DC-link capacitor; consequently, reducing the sudden change in currents during the capacitor charging cycle, which leads to the reduction of input current distortion, but at the same time, increase the voltage ripples on the DC-link capacitor [7].

When developing any solution to mitigate harmonics, there are number of factors that must be taken into account, such as size, simplicity, cost, and effectiveness [8]. Many solutions have been used to reduce harmonics and the most common solutions are line reactors, dc reactors, multi pulse driving, passive filters, active filter. Different types of chopper circuits have been used in the DC-link for that purpose. One type is Boost chopper proposed to adjust the fundamental portion of the current and eliminate harmonics. The importance of this method stems from the fact that it maintains a constant fundamental component value during different operation conditions, which is appropriate for motor drive applications [9]. Another type is the buck-boost chopper that has been used in DC-link and has many advantages. This chopper consists of a reactor, two transistors Q1, Q2, two diodes D1, D2 and a capacitor. Although the utilizing of buck-boost chopper in DC-link decreases the reactors’ size, the load current in this case will pass along two switches or even more causing the conductive losses of drive systems to be a bit higher than in the conventional three phase AC drive systems [10].

Four-quadrant chopper was used instead of buck-boost chopper for power quality improvement purposes. This chopper operates like a hybrid (serial-parallel) filter that passes exclusively currents with high frequencies; consequently, eliminates oscillations in DC-link voltage and currents [11]. Different schemes of choppers with reactors and capacitors have been used, and that led to the reduction of both source currents and output voltages harmonic distortion. The small mass and low weight of this chopper makes t appropriate for integration with motors in VSD systems and with other existing components and circuits. It is necessary to modify the schemes of these choppers in order to make it more suitable for different levels of voltages and operation patterns.

A configuration of Modular Multilevel Converter (MMC) using Dual Active Bridge (DAB) for medium voltage drive applications is proposed to overcome some issues that are familiar in MMC at low frequency operation mode. These problems are the circular current and the capacitor ripples. The scheme demands considerably less number of capacitors and is also appropriate for power flowing bi-directionally, which guarantees regeneration [12]. An isolated bi-directional DC-DC converter with an incorporated cascade scheme is suggested for a localized charge system equability. The integrated cascade structure make it work continuously and increase the equalization current [13]. A mathematical model of the drive system operation depending on asynchronously operating rectifier cascade and robust motor excitation was proposed to study and analyse the harmonic content of the drive power characteristics. The characteristics of control patterns of the inverter switches are taken into account. The developed model is the key for the analysis and prediction of the potential emergency cases occurrences in the electric part of the drive and the loss of control of the inverter switches [14].

In this study, a cascade five-level four quadrant chopper scheme has been used instead of three-level full bridge four quadrant chopper scheme and its influence on time response of the studied system has been tasted and analyzed. The proposed scheme comprises two three-level full bridge four quadrant chopper schemes that are connected in series to avoid the transistors’ failure probability resulting from high voltage drop on these transistors as shown in section 1. Both of four quadrant chopper and cascade five level four quadrant chopper have been studied in detail in section 2; studied system and control strategy are shown in section 3. The mathematical model of studied system is illustrated in section 4. Results and discussion of the studied system are demonstrated in section 5. Finally, the conclusions and recommendations drawn from this work, in addition to references are shown in sections 6, 7 respectively.

## 2 Five-level cascade four-quadrant chopper

The four-quadrant chopper is considered one of the most important configurations used for DC motors driving, in addition, it has great potentials in regulating the voltage and current of the DC circuit under different operation conditions.

### 2.1 Four – quadrant chopper operational principles

**Figure 1:**
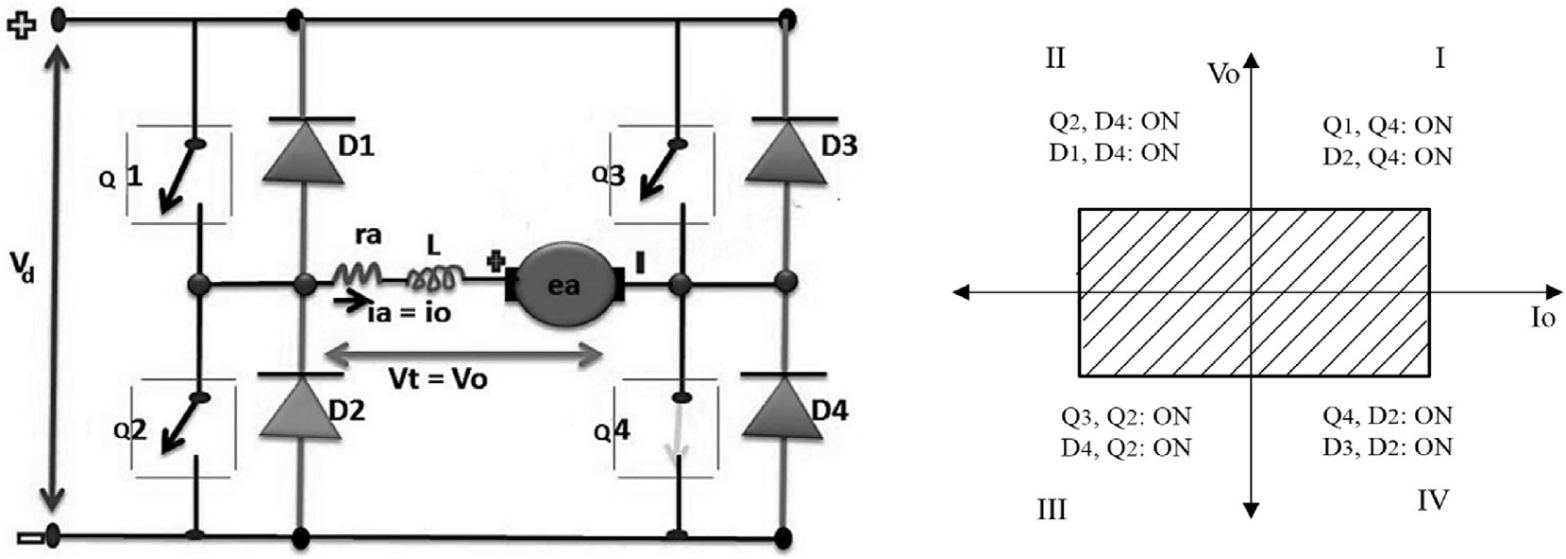
Four quadrant chopper and its operation mode.

Four quadrant chopper is shown in figure (1). The operational principle of the chopper is described as follows:

In the first quadrant, switch Q_4_ is always on and switch Q_1_ is changing between (on-off) mode. When switch Q_1_ is on, the current will pass through V-Q_1_- R- L- E- Q_4_, and by the time switch Q_1_ is off current will pass through R- L- E- Q_4_- D_2_. The chopper acts like a step down chopper in this quadrant, and both V_o_ and I_o_ are positive. In the second quadrant, the switch Q_2_ is changing between (on-off) states, and the other switches (Q_1_-Q_3_-Q_4_) are always off. The inductor L stores power by the time the transistor Q_2_ is turned on. The current will pass through E- L- R- Q_2_- D_4_. In second quadrant, Voltage has positive polarity and current has negative polarity, and the chopper acts like a step up chopper. In the third quadrant, Q_3_ switches between on and off modes, whereas Q_1_ is always off and Q_2_ is always on. The current will pass through Q_2_, D_4_ when Q_3_ is off. The chopper works as a step down chopper in this quadrant and both voltage and current have negative polarity. In the fourth quadrant, the switch Q_4_ changes whereas other switches are always off. The inductor L will store power when Q_4_ is on and current will pass through Q_4_- D_2_- L- E. When Q_4_ is off, current will pass through D_2_-D_3_. The polarity of voltage is negative, while the polarity of current is positive. In this quadrant, the chopper works as a boost chopper [15].

### 2.2 Cascade five-level four quadrant chopper

Recently, Multilevel converters have been a main concern of most researchers in electrical drive and power electronics field because of their various advantages when compared with conventional converters used in the same fields. These converters work at high and low sampling frequencies and give currents and voltages with low harmonic content, low voltage noise, and low electromagnetic interference resulted from semi-conductor elements used in these converters [16]. On the other hand. These converters have many disadvantages, such as the large number of used semi-conductors and drive circuits, which complicates the overall system and increases its costs [17]. Cascade multi-leveled converters can be easily integrated with individual converters, which simplifies the industrial production process of these converters. Multi-level converters can be classified into different categories including: cascade H-bridge multi-level converters (CHMLC), flying capacitors multi-leveled converters (FCMLC), and diodes clamped multi-level converters (DCMLC) and each of these converters has its own structure and special features [18].

**Figure 2:**
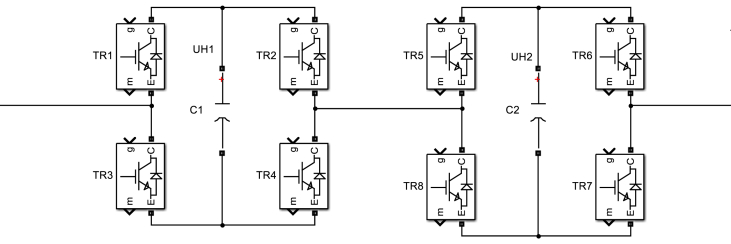
Five-level cascade four quadrant chopper.

Five levels four-quadrant chopper consists of two H-bridges four quadrant choppers, as shown in figure (2), each of these bridges consists of four pairs (transistor-diode) and each bridge produces three different output voltages: 0, +Vdc, -Vdc by operating four transistors TR1, TR2, TR3, TR4 using different operation patterns. In order to obtain +Vdc voltage at each chopper output, transistors TR1 and TR4 must work together and in order to obtain voltage –Vdc, both transistors TR2 and TR3 must work together.

The two choppers are connected in series. Thus, we obtain a chopper with five levels: -2Vdc, -Vdc, 0, + Vdc, + 2Vdc. The total voltage is the sum of individual choppers output voltages as they are connected in series. The output voltage can be expressed in Eq. (2).

(2) $$U_{N}=U_{H1}+U_{H2}$$

where:

*U*_*H1*_: first chopper capacitor voltage.

*U*_*H2*_: second chopper capacitor voltage.

*U*_*N*_: chopper output voltage.

## 3 Studied system and control strategy

The studied system is a medium power variable speed drive system composed of an electrical network, six pulses full bridge diode rectifier, and DC-link with five level cascade four-quadrant chopper integrated with this system to enhance the power quality of the system. The inverter with the motor was replaced with an effective load in order to simplify the study.

There are many modification techniques and control methods in multi-level converters such as; sinusoidal pulse width modulation (SPWM), selective harmonic elimination (SHE), space vector modulation (SVM), and more [19]. The simplest and most common modulation technique is SPWM technique that generates transistor's gates pulses by comparing a sinusoidal signal with triangle-shaped carrier [20]. This technique utilizes (N-1) carrier signal to obtain (N) level voltage on the converter output [21].

**Figure 3:**
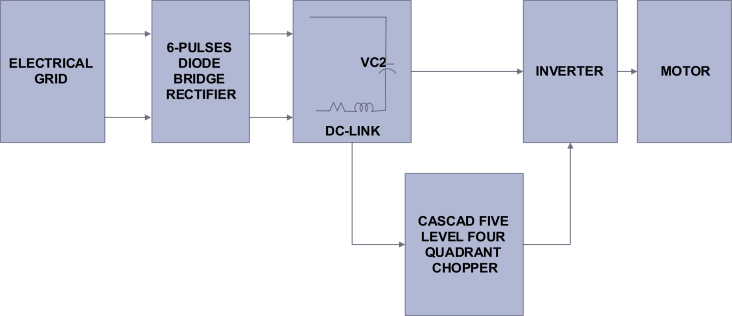
Studied system.

**Figure 4:**
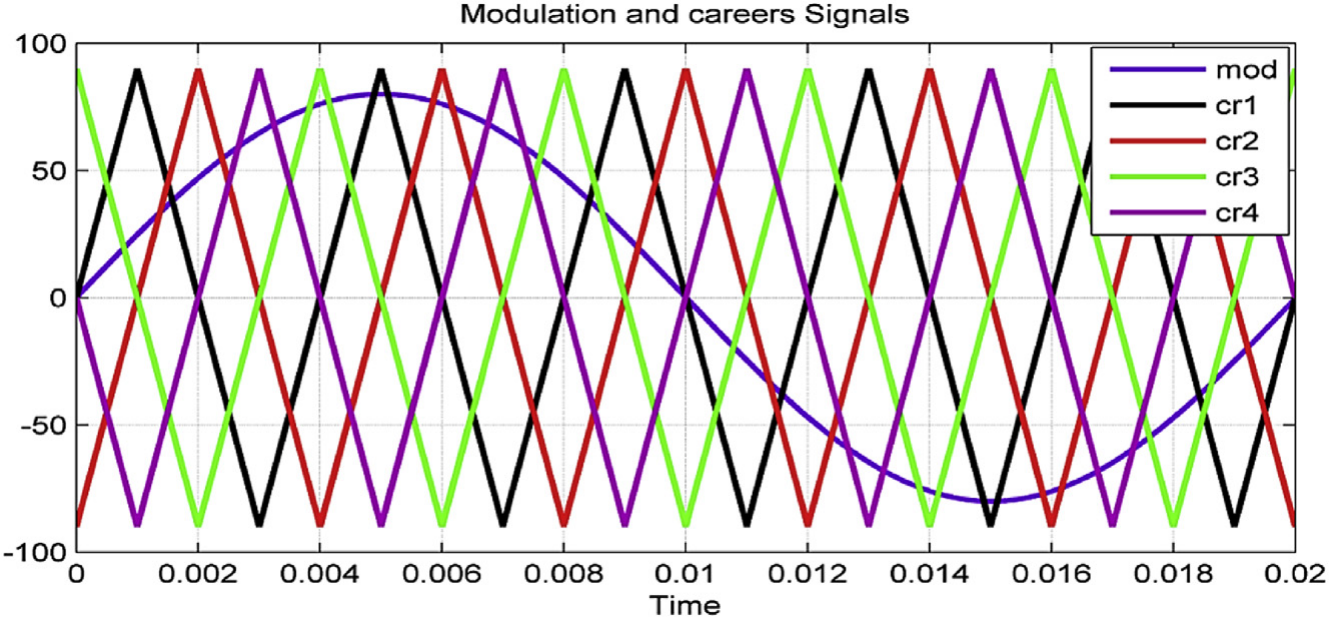
Modulation and careers signals.

To obtain a five-level output voltage on the chopper output, we need four carrier signals (triangle-shaped signals). All triangle-shaped signals have the same amplitude and frequency with a phase shift between every two adjacent signals. This shift can be determined according to Eq. (3) as follow [21].

(3) $$\text{ϕ}=\frac{360}{m-1}$$

where ϕ is the phase shift between two adjacent signals, m is the modulation signal. Gate pulses are obtained by comparing the modulation and the carrier signals. Therefore, for a five-level modulation, five career signals with a phase shift of (-90) degree between adjacent careers are needed [22]. In this case, the phase shift is illustrated in figure (3) and can be expressed as below: (see Figure 4)

$$\begin{array}{cccc} {\text{U}}_{Cr1}=0 & {\text{U}}_{Cr2}=90 & {\text{U}}_{Cr1}=-180 & {\text{U}}_{Cr2}=-270 \end{array}$$

**Figure 5:**
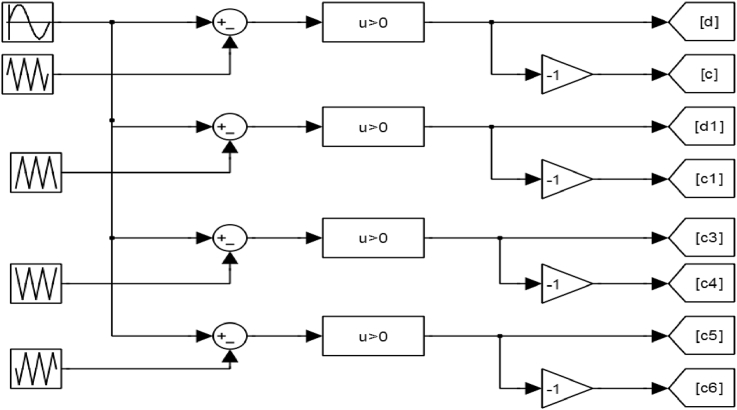
Control scheme of the cascade transistor chopper.

When Um is greater than U_cr1_ and U_cr2_, pulses are given to the gates of upper transistors T_1_, T_5_, and thus these switches will work. When U_m_ is less than U_cr1_ and U_cr2_, pulses are given to the gates of right transistors T_2_, T_6_ (the right part of the bridge) [23]. Lower transistors are complementary to upper transistors. The output voltage of H1 bridge range between 0 and +E at positive pulse half of the signal, and between zero and –E at negative pulse half of the signal, control scheme for transistor chopper is given in figure (5).

Table (1) shows operation patterns of five level cascade four-quadrant chopper. The frequency modulation factor is defined as the ratio between carrier signal frequency and modulation signal frequency:

(4) $$mf=\frac{fc}{fm}$$

where.

**Table 1:** Operation patterns of different types of five level cascade four-quadrant chopper.

Output voltage	Transistors state				H1 cell voltage	H2 cell voltage
U_an_	T_11_	T_21_	T_12_	T_22_	U_H1_	U_H2_
2E	1	0	1	0	E	E
E	1	0	1	1	E	0
	1	0	0	0	E	0
	1	1	1	0	0	E
	0	0	1	0	0	E
0	0	0	0	0	0	0
	0	0	1	1	0	0
	1	1	0	0	0	0
	1	1	1	1	0	0
	1	0	0	1	0	0
	0	1	1	0	E	-E
-E	0	1	1	1	-E	0
	0	1	0	0	-E	0
	1	1	0	1	0	-E
	0	0	0	1	0	-E
-2E	0	1	0	1	-E	-E

mf: The frequency modulation factor.

fc: The carrier signal frequency.

fm: The modulation signal frequency.

The coefficient of amplitude modulation is given as the ratio between the amplitude of modulation signal and the amplitude of carrier signal:

(5) $$ma=\frac{vm}{vc}$$

where:

*ma*: The coefficient of amplitude modulation.

*vm*: The coefficient of amplitude modulation.

*vc*: The carrier signal amplitude.

The chopper switching frequency can be expressed in the following equation:

(6) finv=(m−1).fc

where *finv* is the switching frequency.

**Figure 6:**
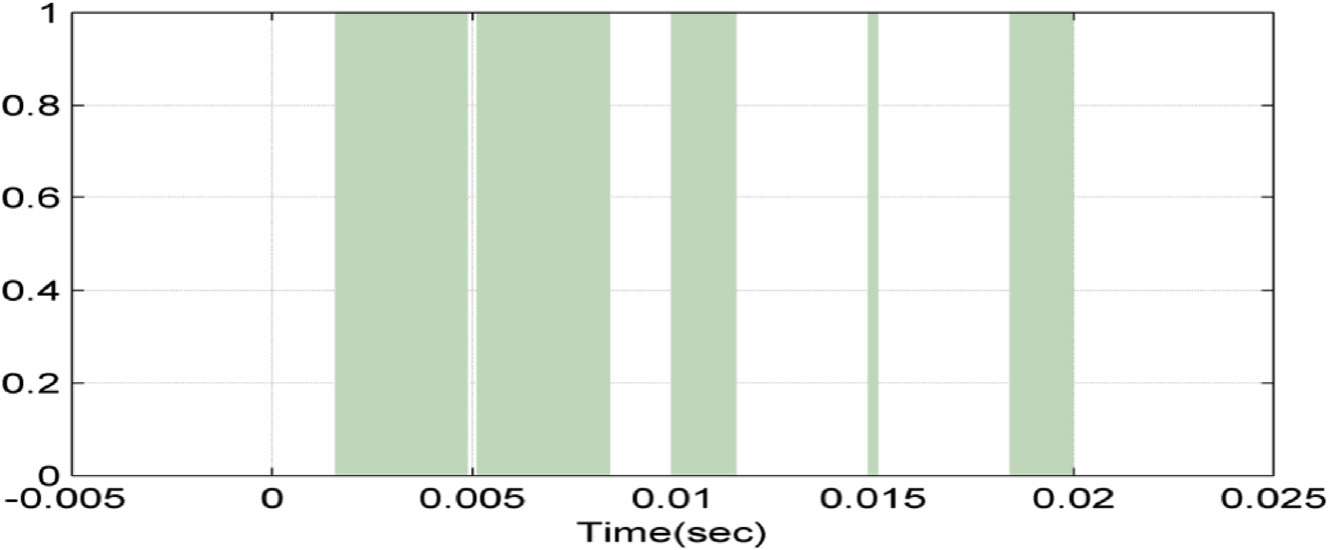
TR_1_ gate signal.

**Figure 7:**
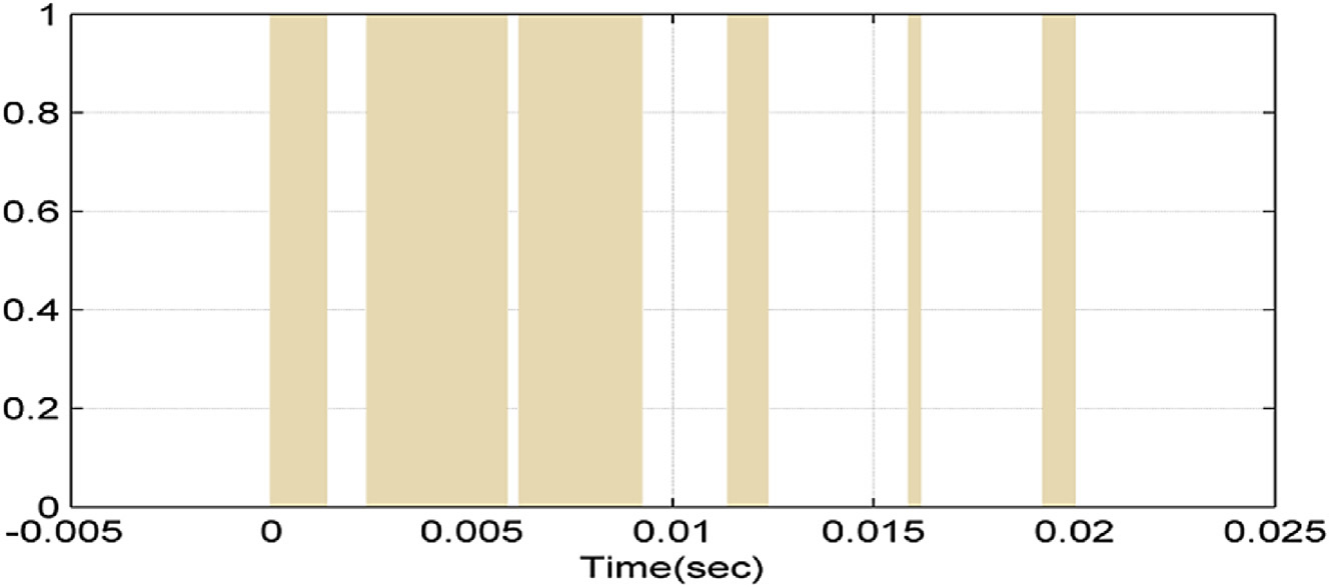
TR_2_ gate signal.

**Figure 8:**
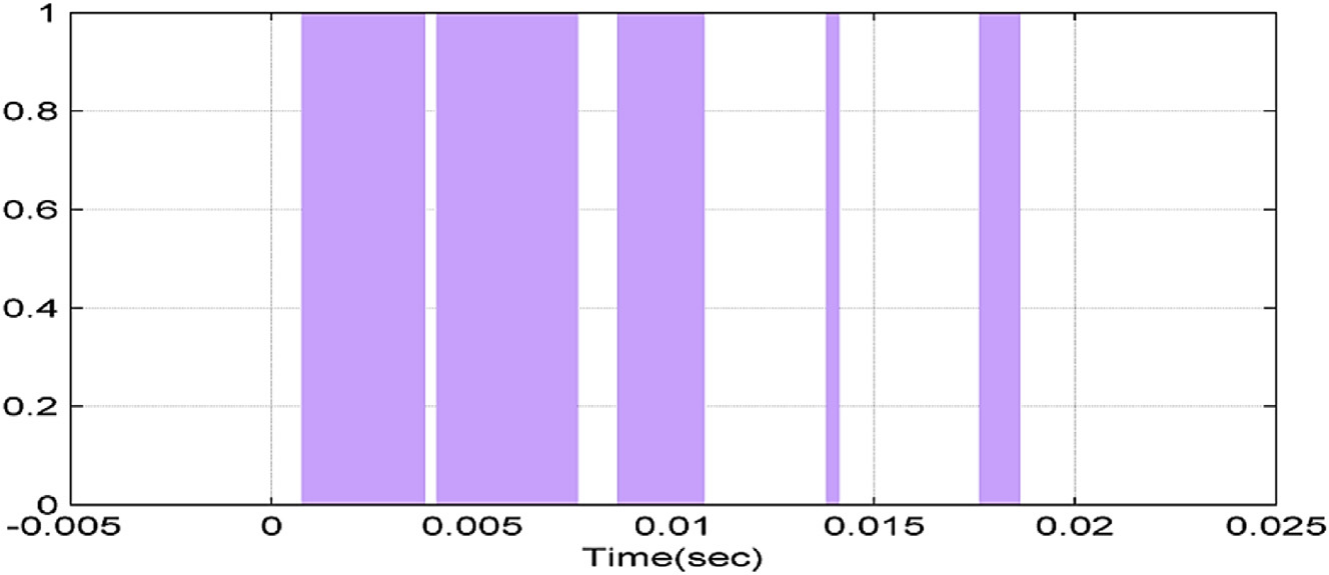
TR5 gate signal.

**Figure 9:**
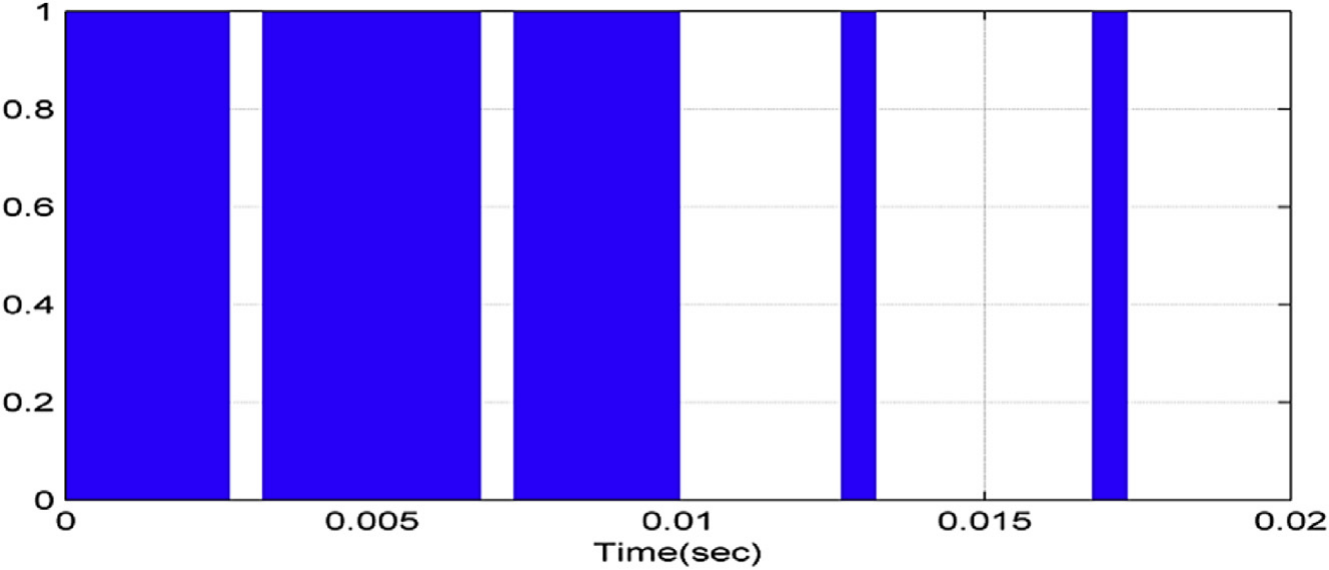
TR6 gate signal.

Figures 6, 7, 8, and 9 show gate signals of chopper transistors.

## 4 Mathematical model

Input voltages of the studied system are given in Eqs. (7), (8), and (9):

(7) $$U_{1}=U_{\max}.\cos\left(wt\right)$$

(8) $$U_{2}=U_{\max}.\cos\left(wt-\theta \right)$$

(9) $$U_{3}=U_{\max}.\cos\left(wt+\theta \right)$$

First, voltage passes through rectifying process. Rectifier Output voltage is the average value that can be defined by Eqs. (10) and (11):

(10) $$U_{d}=\frac{1}{\frac{2\pi }{6}}\cdot \int_{\frac{-\pi }{6}}^{\frac{\pi }{6}}u_{\max}.\cos\left(wt\right)\cdot d\left(\omega t\right)=\frac{1}{\frac{2\pi }{6}}\cdot \int_{\frac{-\pi }{6}}^{\frac{\pi }{6}}\sqrt{3}\cdot u_{\max}.\cos\left(wt\right)\cdot d\left(\omega t\right)$$

(11) $$U_{d}=2.34\cdot U_{1}$$

The output voltage and current of the four-quadrant chopper are given in Eqs. (12), (13), (14), and (15):

(12) $$a=t_{e}/T$$

(13) $$T=t_{e}+t_{a}$$

(14) $$U_{M}=a\cdot U_{d}-\left(1-a\right)\cdot U_{d}=\left(2\cdot a-1\right)\cdot U_{d}$$

(15) $$I_{M}=\left(1/Z_{chopper}\right)\cdot \left(2a-1\right)\cdot U_{d}-U_{C}$$

where:

te: the operating time of T_1_, T_3_.

ta: the operating time of T_2_, T_4_.

a: the duty cycle.

Fast Fourier analysis of the inverter output voltages is given as:

(16) $$V_{ab}=\sum_{n-1,3,5,....}^{\infty }\frac{4U_{d}}{n\pi }.\cos\left(\frac{n\pi }{6}\right).\sin\;n\left(\omega t+\frac{\pi }{6}\right)$$

(17) $$V_{bc}=\sum_{n-1,3,5,....}^{\infty }\frac{4U_{d}}{n\pi }.\cos\left(\frac{n\pi }{6}\right).\sin\;n\left(\omega t-\frac{\pi }{2}\right)$$

(18) $$V_{ca}=\sum_{n-1,3,5,....}^{\infty }\frac{4U_{d}}{n\pi }.\cos\left(\frac{n\pi }{6}\right).\sin\;n\left(\omega t-\frac{7\pi }{6}\right)$$

(19) $$V_{L}={\left[\frac{2}{2\pi }.\int_{0}^{\frac{2\pi }{3}}U_{d}^{2}.d\left(\omega t\right)\right]}^{1/2}$$

## 5 Results and discussion

Both of normal and transient operation states were studied in a medium power variable-speed drive system using the proposed chopper. The results are discussed in detail in both cases as listed below.

### 5.1 Normal operation state of the studied system

The time response of the VSD system with the cascade five – level four-quadrant chopper integrated into DC-link, is studied and compared with the time response of the same system with three-level four-quadrant chopper.

**Figure 10:**
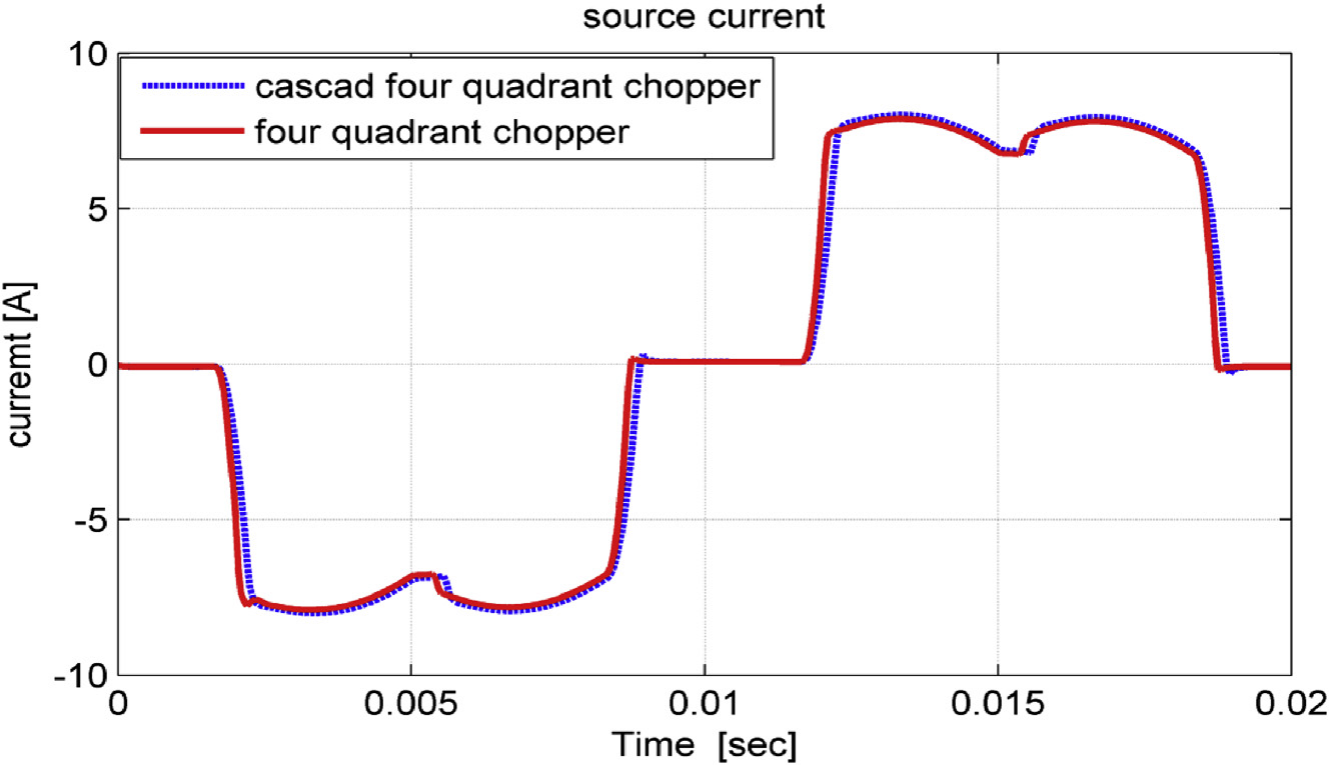
Grid currents.

Grid currents in modern industrial facilities are slightly changed when replacing three-level chopper with five-level chopper as illustrated in figure (10). Moreover, the THD% factor of source current is reduced from (26.7%) when using three-level chopper to (26.2%) when using five-level, which demonstrates the little effect on the input currents when replacing the four-quadrant chopper by cascade chopper.

Cascade five-level four-quadrant chopper acts like hybrid active filter that passes high frequency components and allows residual current to pass to the load.

**Figure 11:**
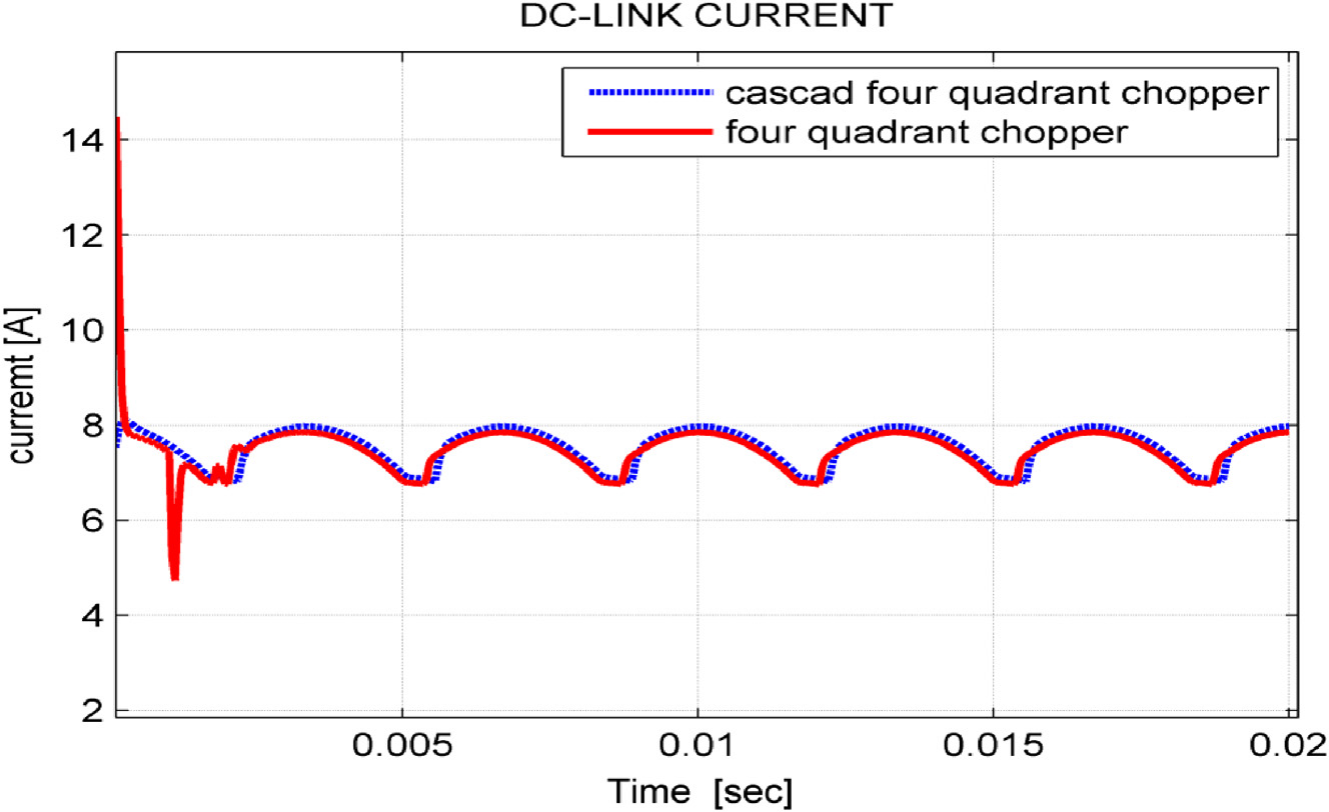
dc – link current.

Figure (11) shows DC-link current for the proposed system when using both choppers mentioned above. It is noticed that cascade chopper eliminates the current surge in transient response and slightly increases the DC-link current.

**Figure 12:**
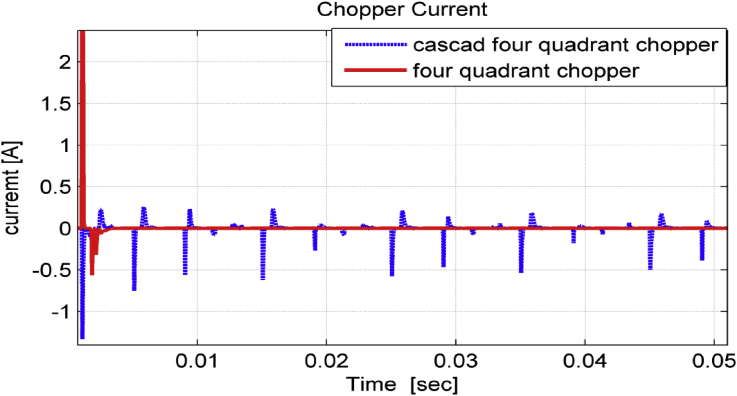
Chopper current.

**Figure 13:**
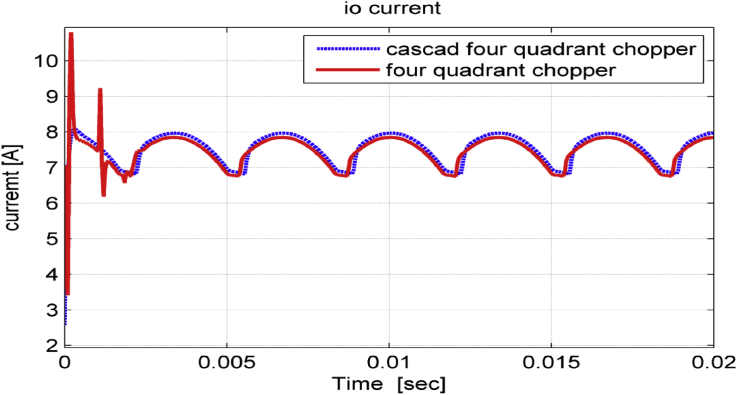
Load current.

Figure (12) shows a comparison between the two choppers currents. It was observed that cascade chopper filtered the distortion components of the Dc-link current better than four-quadrant chopper did. Four-quadrant chopper works only in steady state, whereas cascade chopper works in both transient and steady states. This filtering performance has a good influence on load current (inverter current) and makes it smoother as figure (13) illustrates.

**Figure 14:**
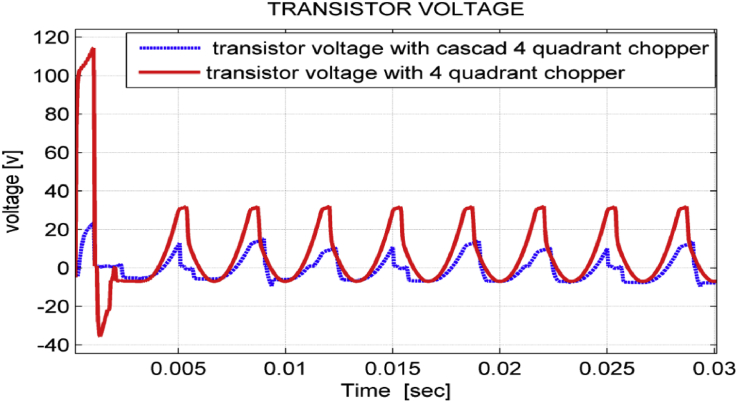
Choppers transistor voltages.

One purpose of the proposed work is to reduce the voltage drop on the electronic elements existed in the chopper circuit in order to avoid the risk of collapse, and the consequent damage and losses in the system. The voltage drop on both four-quadrant chopper and cascade chopper transistors is shown in figure (14). Peak value of voltage in transient state decreases from 115(V) when using four-quadrant chopper to 20(V) when using cascade chopper, while it decreases from 35(V) when using four-quadrant chopper to 15(V) when using cascade chopper in steady state.

### 5.2 Dynamic operation state of the studied system

In order to test the dynamic performance of the studied system, A change has been applied to the system. The proposed change is applying a sudden change on the source voltage by increasing this voltage for a specific period of time. The considered voltage source is a variable voltage source with a linear voltage amplitude of 400V, 0° phase angle, and 50Hz frequency. The applied change is a step change on the considered voltage source with a value of 1.2PU starting at 0.044 s, which equals 72 angle of the third cycle, and ending at 0.08 s.

**Figure 15:**
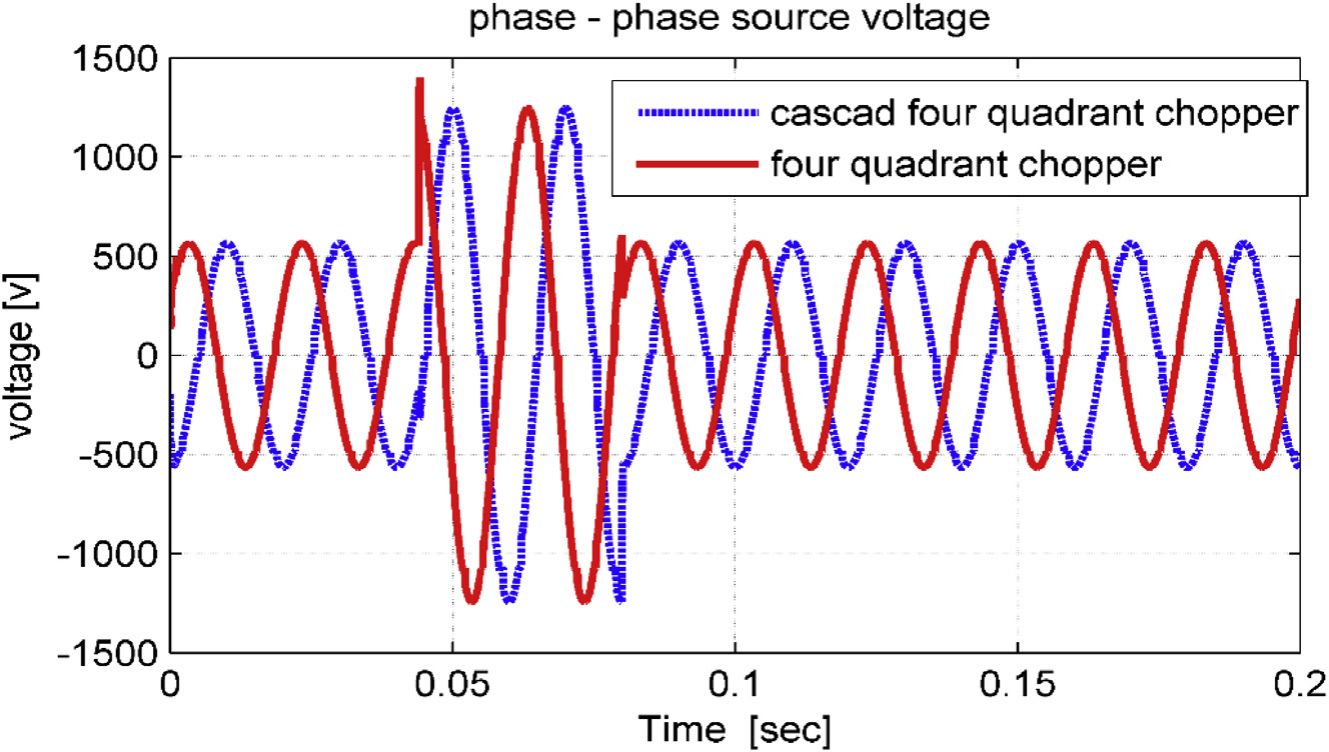
Phase – phase source voltage in transient state.

**Figure 16:**
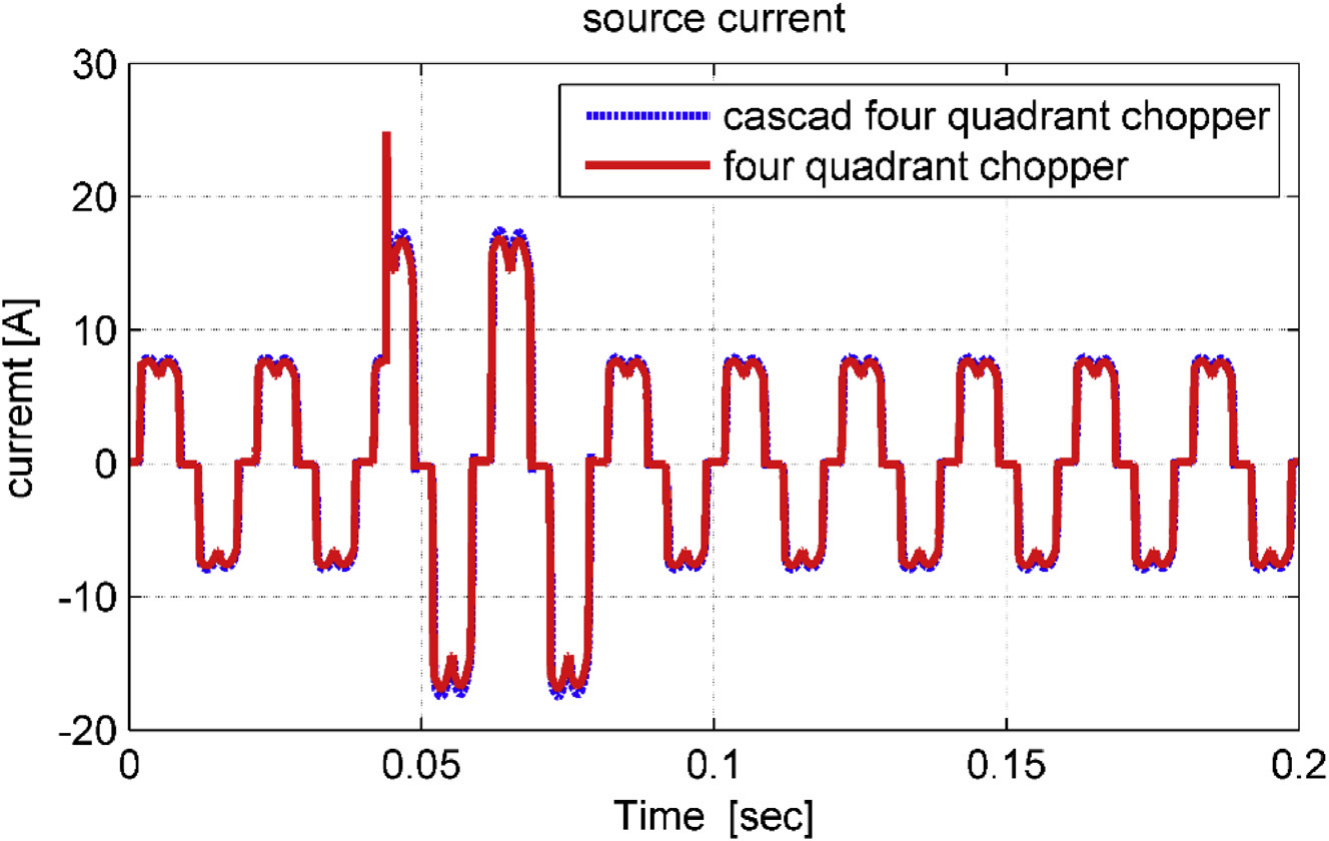
Source current in transient state.

**Figure 17:**
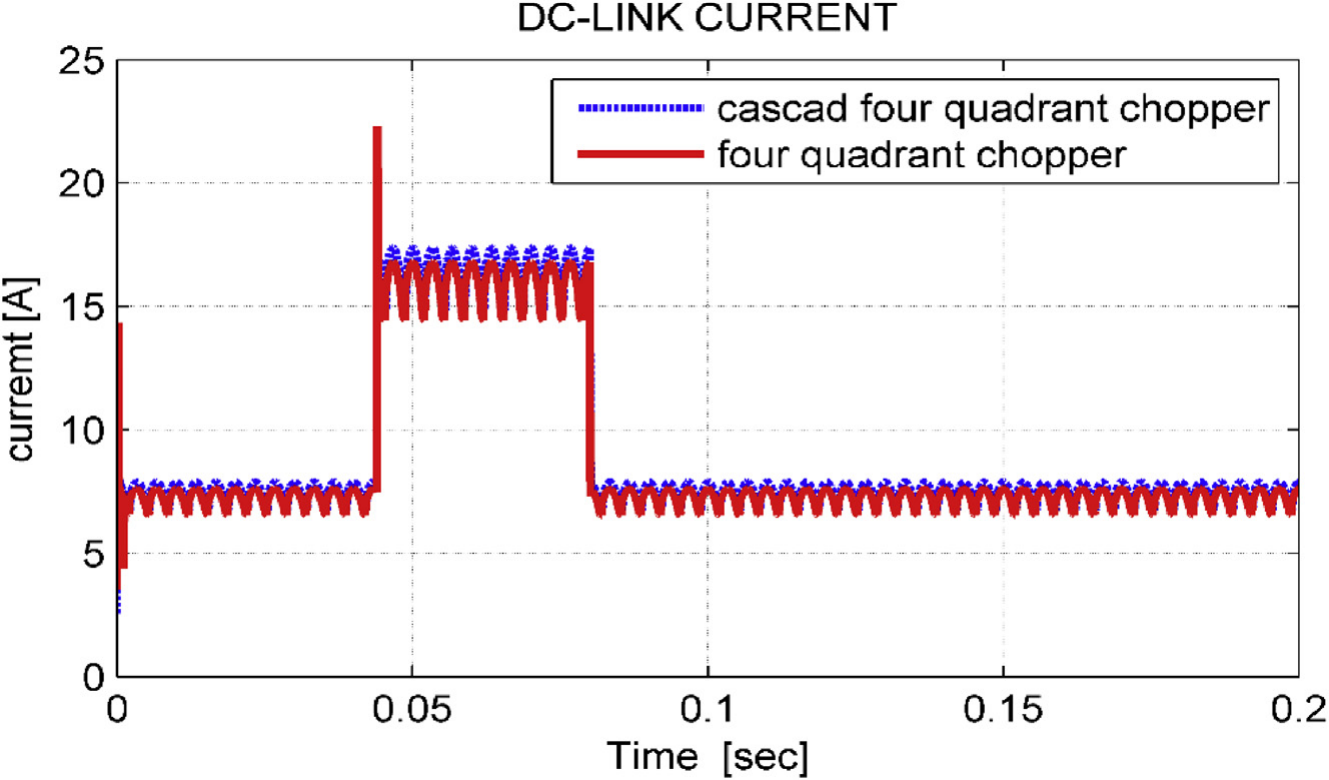
dc-link current in transient state.

As noticed in Figures 15, 16, and 17, the dynamic performance of the studied drive system is smooth when performing a sudden change on source voltage. The system gives better transient behavior with cascade five-level four-quadrant chopper scheme than with three-level four-quadrant chopper scheme. To compare the proposed study with other studies used to improve power quality in industrial facilities, Table 2 was organized [20, 21].

**Table 2:** Comparing between different schemes used to power quality improvement.

Way	Tow level Fixed Hysteresis band control [24]	Fuzzy logic control strategy [24]	Three level SPWM Four-quadrant chopper	Cascade five-level four-quadrant chopper
THDI	29.04%	27.02%	26.7%	26.2%
THDV	4.06%	3.56%	2.54%	2.43%
RF	0.202	0.198	0.198	0.196

It is notice from comparison in Table 2 that the performance specifications of the studied system with suggested chopper scheme are improved. Both of current and voltage at the proposed system input, achieved a minimum THD% factor and RF factor values when compared with other studied ways.

## 6 Conclusions

The wide use of VSD systems has improved the quality of life by enhancing the various applications that occupy most of daily life aspects. VSD systems can be a double-edge sward as these systems can have benefits and drawbacks at the same time. Since VSD systems depend on power electronics and nonlinear elements, using these system can come with many issues, such as power quality problems, switching losses, and EMI problems. This study is concerned with improving the performance of medium voltage VSD system by developing the DC-link circuit in this system. The study proposed merging modern cascade converters instead of conventional converters in DC-link circuit. To perform the study, a Three-level H-bridge four-quadrant chopper conventional scheme has been developed into modern cascade five-level H-bridge four-quadrant scheme. Results showed that using the proposed scheme has improved the system time response in both normal and dynamic operation states and reduced voltage drop on chopper transistors when compared to the conventional scheme. In addition, the proposed scheme outperformed different types of techniques in reducing THD% factor of both source current and DC-link voltage and also reducing RF factor of DC-link current. As a future work, it is recommended to use flying capacitors multi-level chopper (FCMLC), or diodes clamped multi-level chopper (DCMLC), and study transient states under different operation conditions in order to determine properties and effects of the used switches on the system's performance in terms of reducing oscillations and switching losses.

## Declarations

### Author contribution statement

Safwan Nadweh, Ola Khaddam, Ghassan Hayek, Bassan Atyieh & Hassan Haes Alhelou: Conceived and designed the experiments; Performed the experiments; Analyzed and interpreted the data; Wrote the paper.

### Funding statement

This research did not receive any specific grant from funding agencies in the public, commercial, or not-for-profit sectors.

### Competing interest statement

The authors declare no conflict of interest.

### Additional information

No additional information is available for this paper.
